# Supplementary material for: Rescue Therapy with Furazolidone in Patients with at Least Five Eradication Treatment Failures and Multi-Resistant H. pylori infection
Source: Antibiotics (Basel). 2021 Aug 24;10(9):1028. doi: 10.3390/antibiotics10091028 (PMC8467492; doi:10.3390/antibiotics10091028)
Supplement: Supplementary file 1 [file antibiotics-10-01028-s001.zip › antibiotics-1329531-supplementary.pdf]

Supplementary materials

Case report form used for collection of information on adverse events

Has the patient reported any adverse events?	◯ Yes<br>◯ No
Common Adverse Events	
Select those reported	☐ Dysgeusia<br>☐ Diarrhoea<br>☐ Nausea<br>☐ Vomits<br>☐ Dyspepsia<br>☐ Heartburn<br>☐ Abdominal Pain<br>☐ Asthenia<br>☐ Anorexia<br>☐ Others
Other Adverse Events	
Number of other Adverse events	◯ 1 ◯ 2 ◯ 3 ◯ 4<br>◯ 5
Specify other adverse event 1	________________________
Specify other adverse event 2	________________________
Specify other adverse event 3	________________________
Specify other adverse event 4	________________________
Specify other adverse event 5	________________________

Intensity of adverse events *

	Mild	Moderate	Severe
Dysgeusia (Metallic taste/taste alterations)	◯	◯	◯
Diarrhoea	◯	◯	◯
Nausea	◯	◯	◯
Vomits	◯	◯	◯
Dyspepsia	◯	◯	◯
Heartburn	◯	◯	◯
Abdominal pain	◯	◯	◯
Asthenia	◯	◯	◯
Anorexia	◯	◯	◯
[otherae1]	◯	◯	◯
[otherae2]	◯	◯	◯
[otherae3]	◯	◯	◯
[otherae4]	◯	◯	◯
[otherae5]	◯	◯	◯

*Mild: as not interfering with daily routine, Moderate: as affecting daily routine, Severe: as not allowing normal daily routine

Length of adverse events

	1 day	2 days	3 days	4 days	5 days	6 days	7 days	8-14 days	15-30 days	31-60 days
Dysgeusia	○	○	○	○	○	○	○	○	○	○
Diarrhoea	○	○	○	○	○	○	○	○	○	○
Nausea	○	○	○	○	○	○	○	○	○	○
Vomits	○	○	○	○	○	○	○	○	○	○
Dyspepsia	○	○	○	○	○	○	○	○	○	○
Heartburn	○	○	○	○	○	○	○	○	○	○
Abdominal pain	○	○	○	○	○	○	○	○	○	○
Asthenia	○	○	○	○	○	○	○	○	○	○
Anorexia	○	○	○	○	○	○	○	○	○	○
[otherae1]	○	○	○	○	○	○	○	○	○	○
[otherae2]	○	○	○	○	○	○	○	○	○	○
[otherae3]	○	○	○	○	○	○	○	○	○	○
[otherae4]	○	○	○	○	○	○	○	○	○	○
[otherae5]	○	○	○	○	○	○	○	○	○	○

Serious Adverse Events

Has the patient suffered from a serious adverse event?<br>(Serious Adverse Event: leading to patient's hospitalization, disability, or death, or to birth defects on pregnant patients	○ Yes<br>○ No
Describe the Serious Adverse Event<br>(Type, length, intensity, duration of the event, outcome of the event)	________________________

Figure S1. Adverse effects: Specific questionnaire.
